# Targeted insertion of regulatory elements enables translational enhancement in rice

**DOI:** 10.3389/fpls.2023.1134209

**Published:** 2023-03-31

**Authors:** Rundong Shen, Qi Yao, Dating Zhong, Xuening Zhang, Xinbo Li, Xuesong Cao, Chao Dong, Yifu Tian, Jian-Kang Zhu, Yuming Lu

**Affiliations:** ^1^ Shanghai Center for Plant Stress Biology, Center for Excellence in Molecular Plant Sciences, Chinese Academy of Sciences, Shanghai, China; ^2^ Center for Advanced Bioindustry Technologies, and Institute of Crop Sciences, Chinese Academy of Agricultural Sciences, Beijing, China; ^3^ Hainan Yazhou Bay Seed Lab, Sanya, Hainan, China; ^4^ Shanghai Collaborative Innovation Center of Agri-Seeds, Joint Center for Single Cell Biology, School of Agriculture and Biology, Shanghai Jiao Tong University, Shanghai, China; ^5^ Institute of Advanced Biotechnology, and School of Life Sciences, Southern University of Science and Technology, Shenzhen, China

**Keywords:** translational regulation, enhancer, knock-in, rice, breeding

## Abstract

*In-locus* editing of agronomically-important genes to optimize their spatiotemporal expression is becoming an important breeding approach. Compared to intensive studies on mRNA transcription, manipulating protein translation by genome editing has not been well exploited. Here, we found that precise knock-in of a regulating element into the 5’UTR of a target gene could efficiently increase its protein abundance in rice. We firstly screened a translational enhancer (AMVE) from alfalfa mosaic virus using protoplast-based luciferase assays with an 8.5-folds enhancement. Then the chemically modified donor of AMVE was synthesized and targeted inserted into the 5’UTRs of two genes (*WRKY71* and *SKC1*) using CRISPR/Cas9. Following the *in-locus* AMVE knock-in, we observed up to a 2.8-fold increase in the amount of WRKY71 protein. Notably, editing of *SKC1*, a sodium transporter, significantly increased salt tolerance in T2 seedlings, indicating the expected regulation of AMVE knock-in. These data demonstrated the feasibility of such *in-locus* editing to enhance protein expression, providing a new approach to manipulating protein translation for crop breeding.

## Introduction

Over the past decades, transgene is one of the most important technologies to modify gene expression and has been widely used in countless research and breeding applications. With the development of CRISPR/Cas technology, optimizing the spatiotemporal expression of agronomically important genes through genome editing is gradually becoming a trend in molecular breeding. Currently, it is mainly achieved by large-scale editing of promoters to randomly alter the transcript level of target genes, and there have been several very promising application studies (Rodriguez-Leal et al., 2017; Shi et al., 2017; Zeng et al., 2020; Wang et al., 2021; Liu et al., 2021a; Song et al., 2022). It is well known that gene expression consists of two parts, transcription and translation. And current research shows that protein translation can also violently regulate protein abundance (Leppek et al., 2018). However, there are few studies on translational regulation in plants, and the use of genome editing to *in-locus* modify gene sequences for translational regulation is rarely reported.

Similar to cis-elements in promoters that regulate transcription, UTRs (untranslated regions) have been proven to greatly affect protein translation (Leppek et al., 2018). For example, upstream open reading frames (uORFs) are typical translational elements that are thought to potentially inhibit the translation of main ORFs. And by editing of uORFs, several studies have successfully increased the protein abundance of target genes without transcriptional perturbation (Xu et al., 2017; Zhang et al., 2018; Liu et al., 2021b). In addition to uORF, many cis-acting RNA elements within the 5’UTR, known as translational enhancers, also contribute to the translational regulation of mRNAs (Leppek et al., 2018). The most extensively used enhancer is the Omega element from the tobacco mosaic virus (TMV). It was widely used to increase protein expression in many transgenic vectors by inserting it into the 5’UTR of target genes (Gallie and Walbot, 1992; Gallie, 2002). And another leading sequence from alfalfa mosaic virus (AMV) has also been used to facilitate protein translation (named AMVE) (Jobling and Gehrke, 1987). In addition, some endogenous sequences of 5’UTRs in plants also could enhance gene expression, such as the 5’UTR of the rice *ADH1* (named ADHE) (Sugio et al., 2008). Apart from the native sequences, some synthetic 5’UTRs (e.g. SynJ, SynM, MsynJ and pSTART) were able to promote transgene expression as enhancers (De Amicis et al., 2007; Kanoria and Burma, 2012). Even under a strong promoter like CaMV 35S, these translational enhancers can improve the transgene expression significantly. Based on the fact of such translational regulations, we hypothesized that targeted knock-in of translational enhancers into the 5’UTR of endogenous genes could increase protein translation, thus providing a new strategy of gene activation with important application value. However, targeted sequence knock-in in plants has been a huge challenge, severely limiting such attempts.

With the development of CRISPR/Cas, the sequence knock-in technologies have made great progresses in plants (Li et al., 2016; Li et al., 2018; Li et al., 2019; Dong et al., 2020; Lu et al., 2020a; Lu et al., 2020b). And we have recently developed an efficient sequence knock-in method in rice using chemically modify donor DNA with frequencies of up to 50% (Lu et al., 2020b). This technological breakthrough makes it possible to knock-in translational enhancers into 5’UTRs. Here, we screened a functional enhancer AMVE and evaluated its feasibility of *in-locus* activation by targeted inserting into 5’UTRs of two genes.

## Materials and methods

### Plant materials and growth condition

The plant materials used in the study were Nipponbare (*Oryza sativa* L. ssp. *Japonica* cv. Nipponbare). The seeds were firstly germinated in Hoagland’s solution and rice seedlings were grown in a growth chamber at 30°C for a 12-hours light period and 28°C for a 12-hours dark period. Two-week-old rice plants were transplanted to the fresh Hoagland’s solution for subsequent treatment. Morphological characteristics were recorded within seven days.

### Plasmid construction

The pDLUC01 and pCBSG032 vectors were stored in our laboratory. The translational enhancer sequences were synthesized and cloned into the digested pDLUC01 vector for protoplast transfection. The target sequences of sgRNA for rice genes were designed using CRISPR-GE and/or CRISPR-P (Liu et al., 2017; Xie et al., 2017) and cloned into the *Bsa*I-digested pCBSG032 vector to construct CRISPR/Cas9 plasmids as previously reported (Lu et al., 2017). Primers used for plasmid construction were listed in **Supplementary Table 3**.

### Protoplast transformation

Rice seedlings grown on MS medium (1/2x, 28°C for 7-10 days) were used for protoplast preparation. The dual-luciferase reporter plasmids with different translational enhancer sequences were transformed into protoplasts, respectively. According to previous described methods, 10 μg of plasmid DNA were transfected into approximately 5 × 10^5^ protoplasts. After a 12-hours incubation at 25°C in dark, protoplasts were harvested by brief centrifugation (100 g for 3 minutes) for subsequent experiments. FLuc/RLuc activity was measured using a Dual-Luciferase Reporter Assay Kit (Vazyme Biotech Co. Ltd, Nanjing, China).

### Generation of knock-in rice

Sequence knock-in rice was produced according to a previous study with some minor modifications (Lu et al., 2020b). Briefly, CRISPR/Cas9 plasmids were firstly prepared using a plasmid Midi-preparation Kit (Tiangen Biotech Co. Ltd, Beijing, China) and diluted to 1 μg/μl. Then, 0.1 pmol CRISPR/Cas9 plasmids were mixed with 2 pmol donor DNA and coated to 3 mg golden particles. Bombardment-mediated transformations were conducted on one-month-old rice calli of Nipponbare with the PDS1000/He particle bombardment system (Bio-Rad, California, USA). The transformed calli were then cultured at 28°C in the dark for 16 hours and transferred onto the selection medium with hygromycin (50 mg/L) for plantlets regeneration (Lu et al., 2017).

### Genotyping

Genomic DNA of the regenerated rice was extracted by CTAB method. Forward and reverse primers were designed within 200 bp of the target site, respectively. The PCR product containing the inserted sequence was larger than that of the wild type. The amplified PCR products were cloned into TA cloning vector pCE2-TA/Blunt-Zero (Vazyme Biotech Co. Ltd, Nanjing, China) for Sanger sequencing and/or directly sequenced using the Hi-TOM method. The PCR primers used for genotyping are listed in **Supplementary Table 3**.

### RNA extraction and qRT-PCR

Total RNA was extracted from the rice protoplasts or young leaves using the Plant Total RNA Extraction Kit (Sango Biotech Co. Ltd, Shanghai, China). Reverse transcription was performed with 1 μg using HiScript cDNA Synthesis Kit (Vazyme Biotech Co. Ltd, Nanjing, China) according to the manufacturer’s instruction. Quantitative PCR (qPCR) was performed using ChamQ SYBR Master Mix (Vazyme Biotech Co. Ltd, Nanjing, China). Each qPCR assay was replicated at least three times. The *RLuc* gene was used as an internal control for rice protoplasts and the rice *Actin1* gene was used as the housekeeping gene for plant samples. The primers were listed in **Supplementary Table 3**.

### Protein extraction and immunoblot analysis

Total proteins were extracted from rice leaves using 2×SDS loading buffer. The protein samples were separated using a 12% SDS-PAGE gel and transferred onto PVDF membranes. Membranes were blocked with 5% skim milk for 1 hour and incubated overnight at 4°C with primary antibodies of rabbit anti-Actin antibody (Sango Biotech Co. Ltd, Shanghai, China, 1:1,000 dilution) or anti-OsWRKY71 antibody (Beijing Protein Innovation Co. Ltd, Beijing, China, 1:1,000 dilution). The membranes were washed three times with TBST and incubated with secondary antibodies for 1 hour at room temperature. The secondary antibody was a goat anti-rabbit antibody conjugated to horseradish peroxidase (Sango Biotech Co. Ltd, Shanghai, China). Protein signals were detected with Clarity Western ECL Substrate (Bio-Rad, California, USA) using ChemiDocXRS imaging system (Bio-Rad, California, USA).

### Bacteria strains and inoculation

*Xoo* strain (PXO99) was grown on PSA plates (10 g/l peptone, 10 g/l sucrose, 1 g/l glutamic acid, 16 g/l bacto-agar, pH 7.0) at 28 °C for 2 days. The bacterial cells were suspended in sterile water to an OD600 0.6 for inoculation. Leaves of rice plants (6-8 weeks old) were inoculated using a leaf-clipping method as previously described. Disease symptoms were assessed 14 days after inoculation by measuring the lesion length.

### Off-target analysis

The CRISPR-P (v.2.0) program was used to predict the potential off-target sites for sgRNAs in rice genome (Liu et al., 2017). Potential off-target sites were PCR amplified from the edited lines and subjected to Sanger sequencing. The PCR primers used for off-target analysis are listed in **Supplementary Table 3**.

## Results

### Screening of translational enhancers for rice

**Figure 1:**
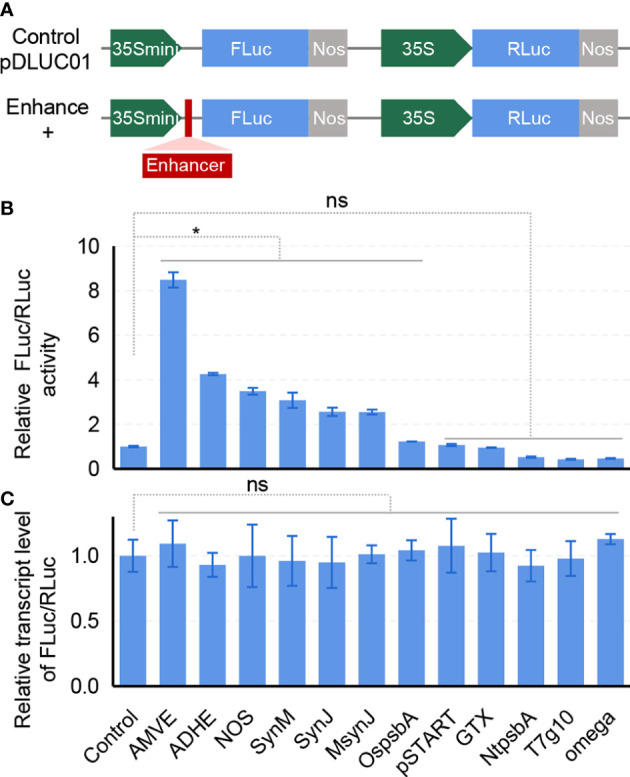
Screening of translational enhancers in rice protoplasts. **(A)** Diagram of the dual-luciferase plasmid pDLUC01. FLuc, firefly luciferase. RLuc, *R. reniformis* luciferase. The insertion site for enhancer sequences was marked as a red box. **(B)** Assessment of enhancers in protoplast-based dual-luciferase assays. RLuc was used as an internal control. **(C)** Transcript levels of FLuc/RLuc in rice protoplasts. **(B, C)** All values represent means ± s.d. of three independent experiments (n = 3). *P < 0.05; ns, no significant difference (two-tailed Student’ s *t* test).

According to previous studies on translational enhancers, twelve candidates were selected, including the virus-derived Omega and AMVE (Jobling and Gehrke, 1987; Gallie and Walbot, 1992; Gallie, 2002), plant-derived ADHE, OspsbA and NtpsbA (Zou et al., 2003; Sugio et al., 2008), and synthetic sequences SynM, SynJ, MsynJ and pSTART (De Amicis et al., 2007; Kanoria and Burma, 2012) (**Supplementary Figure 1B**). To conduct a quick evaluation in rice, a protoplasts-based transient assay was developed. A dual-luciferase reporter (pDLUC01) was constructed to access the effects of these translational enhancers (**Figure 1A**). The firefly luciferase (*FLuc*) was used as the reporter and the *R. reniformis* luciferase (*RLuc*) was used as the internal control. Enhancer sequences were inserted into the 5’UTR of *FLuc* driven by a truncated 35S promoter (35Smini, **Supplementary Figure 1A**). Enhancers were then parallelly assessed in rice protoplasts. The results showed that six of them had enhancement effect, especially AMVE and ADHE, which remarkably increased 8.5 times and 4.3 times on FLuc expression, respectively (**Figure 1B**). As expected, quantitative RT-PCR (qRT-PCR) analysis showed that there was no significant difference in the transcript level of FLuc/RLuc, suggesting that such regulation possibly happened at the translational level without altering mRNA abundance (**Figure 1C**). It could be noticed that the Omega enhancer derived from TMV that was widely used in dicots did not function in rice, indicating a possible specificity of species requirement for such translational enhancers. Accordingly, we chose AMVE and ADHE for subsequent tests.

### Translational enhancement of *WRKY71*

**Figure 2:**
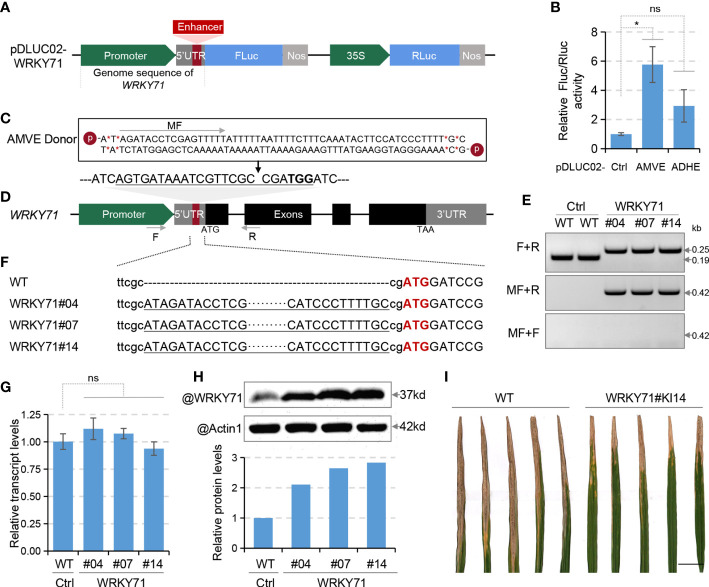
Translational enhancement of *WRKY71*. **(A)** Schematic diagram of dual-luciferase plasmid for *WRKY71* (pDLUC02-WRKY71). **(B)** Assessment of enhancers ADHE and AMVE in protoplast-based dual-luciferase assays. Plasmids pDLUC02-WRKY71 with or without (Ctrl) enhancer sequences were used as reporters. **(C)** The chemically modified donor sequence of AMVE. **(D)** Schematic diagram of *WRKY71* for AMVE knock-in. The sgRNA target of Cas9 is underlined with the protospacer-adjacent motif (PAM) in bold. **(E)** Genotyping of AMVE knock-in mutants on *WRKY71* with PCR. Genomic DNA was amplified using primers flanking target sites (F+R). Forward (middle) and reverse (lower) insertion mutants were identified using primers F+MR and F+MF, respectively. Positions of primers are indicated as arrows in **(C, D)**. **(F)** Sequencing results of AMVE knock-in mutants. Sequences of AMVE were underlined and the start codon of *WRKY71* is in red. **(G)** Transcription levels of *WRKY71* in WT (Ctrl) and T1 mutants were determined using qRT-PCR. The rice *Actin1* gene was used as the internal control. **(H)** Comparison of the protein level of WRKY71 between the WT (Ctrl) and T1 mutants. Actin1 was used as the loading control in the immunoblot assay. The ratio of the gray value (WRKY71/Actin1) was calculated using ImageJ. **(I)** Representative results of lesion length symptoms caused by *Xoo* strains in rice leaves. Photograph was taken two weeks after inoculation with PXO99. Bar, 2 cm. **(B, G)** All values represent means ± s.d. of three independent experiments (n = 3). *P < 0.05; ns, no significant difference (two-tailed Student’ s *t* test).

Unlike the transcriptional regulation that could be easily quantified using qRT-PCR, assessment of translational regulation usually requires antibodies corresponding to the target genes for immunoblot assays. Thus we chose *WRKY71* as the target gene for its commercially available antibody and proven functions in bacterial resistance (Liu et al., 2007; Liu et al., 2016). To do a quick evaluation of AMVE and ADHE on *WRKY71*, the promoter and 5’UTR sequence of *WRKY71* were cloned into pDLUC01 reporter to replace the 35Smin promoter (pDLUC02-WRKY71), and AMVE and ADHE were then inserted into its 5’UTR (**Figure 2A**). Protoplast-based assay indicated that both AMVE and ADHE could increase the FLuc/RLuc activity by 5.8 and 2.9 times, respectively (**Figure 2B**). Thus, AMVE was selected for knock-in.

To generate AMVE knock-in rice, chemically modified donor DNA of AMVE was synthesized according to our previous studies (**Figure 2C**). A sgRNA target within the 5’UTR of *WRKY71* was designed (**Figure 2D**) and a total of 60 T0 rice plants were generated using bombardment-mediated transformation. Among the 11 knock-in plantlets, three T0 plants had a precisely forward and seamless insertion of AMVE and their T1 homozygous were identified and used for evaluations (**Figures 2E, F**; **Supplementary Tables 1**, **4**). Similar to the results in protoplasts, the transcript levels of *WRKY71* in knock-in mutants (WRKY71#04, #07 and #14) remained unchanged (**Figure 2G**), while its protein abundance was increased obviously by about 2.8 times identified with immunoblot assays (**Figure 2H**). Consistent with previous studies (Liu et al., 2007; Liu et al., 2016), up-regulation of *WRKY71* improved the resistance to *Xanthomonas oryzae* (*Xoo*) of bacterial blight (**Figure 2I**). These results demonstrated the feasibility of such translational manipulations *via in-locus* editing.

### Knock-in of AMVE to improve salt tolerance of rice

**Figure 3:**
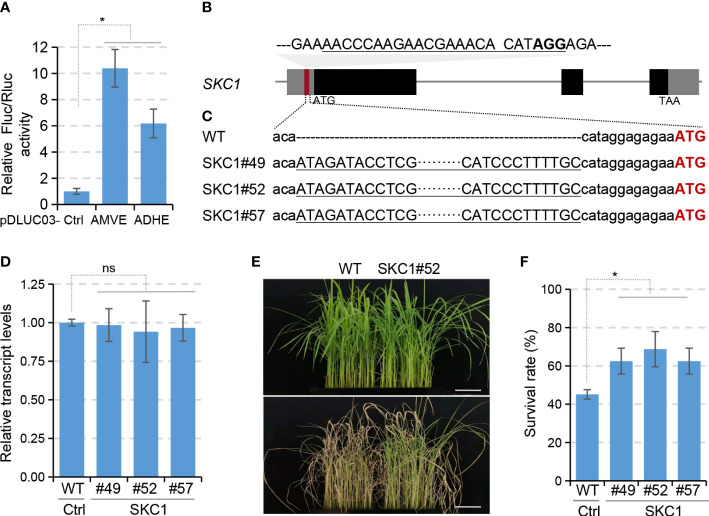
Knock-in of AMVE to improve salt tolerance of rice. **(A)** Assessment of enhancers ADHE and AMVE in protoplast-based dual-luciferase assays. Plasmids pDLUC02-SKC1 with or without (Ctrl) enhancer sequences were used as reporters. **(B)** Schematic diagram of *SKC1* for AMVE knock-in. The sgRNA target of Cas9 is underlined with the PAM in bold. **(C)** Sequencing results of AMVE knock-in mutants. Sequences of AMVE were underlined and the start codon of *SKC1* is in red. **(D)** Transcription levels of *SKC1* in WT (Ctrl) and T2 mutants were determined using qRT-PCR. The rice *Actin1* gene was used as the internal control. **(E, F)** Morphological and survival comparisons of the WT plants and T2 mutants in salt-stress treatment. Four-week-old rice seedlings (upper) were treated with 150 mM NaCl for 7 days (lower). Bar, 5 cm. **(A, D, F)** All values represent means ± s.d. of three independent experiments (n = 3). *P < 0.05; ns, no significant difference (two-tailed Student’ s *t* test).

To further validate the strategy, we selected another important gene *SKC1* that encodes a Na^+^-selective transporter (Ren et al., 2005; Kobayashi et al., 2017; Quan et al., 2017). *In vitro* assays using protoplasts as described above demonstrated that inserting AMVE into 5’UTR of *SKC1* in the luciferase reporter pDLUC02 resulted in a 10.4-folds enhancement (**Figure 3A**), which convinced us to do subsequent gene editing. A sgRNA target of CRISPR/Cas9 was designed in the 5’UTR of *SKC1* (**Figure 3B**) and a total of 119 T0 plants were regenerated *via* biolistic-mediated transformation. We identified 32 targeted insertion mutants from these transgenic lines. Six precise knock-in mutants containing a forward AMVE insert were identified by Sanger sequencing (**Figure 3C** and **Supplementary Table 1**). Unfortunately, our efforts to make SKC1 antibodies failed (**Supplementary Figure 3**). Since previous studies demonstrated that up-regulation of *SKC1 via* transgene can increase the salinity tolerance of rice mutants (Farquharson, 2009), three lines of them (SKC1#49, #52 and #57) were selected directly for salinity-tolerance evaluations.

A population of T2 mutants was generated from homozygotes of their T1 lines. qRT-PCR results showed that the transcript levels of *SKC1* in the three AMVE knock-in mutants were comparable to those in WT (**Figure 3D**). Two-week-old seedlings grown in liquid Hoagland’s solution were then treated with 150 mM NaCl. After 7-days treatment, the three edited mutant lines showed more tolerance to salt stress than WT plants (**Figure 3E**) and gave a much more survival rate (45% vs. 65%, **Figure 3F**). These results were consistent with previous findings (Farquharson, 2009), indicating an up-regulation of *SKC1*. Because the transcript level of *SKC1* remained unchanged, we believed that such an AMVE-mediated up-regulation happened at the translational level, consistent with the results of *WRKY71*. In addition, we observed a genetic segregation of a 1:2:1 for these T1 heterozygotes that followed the Mendel’s law (**Supplementary Table 2**), indicating that both the genotype and phenotype may be passed on to offspring. These results demonstrated that the knock-in of enhancers into 5’UTRs could be useful for plant breeding.

## Discussion and conclusions

Transgene-mediated up-regulation of a target gene is widely used in plant research and breeding that usually driven by a constitutive promoter. Such transcriptional manipulation is currently the main method of gene expression regulation. Now we have entered the era of gene editing, and sequence modification in promoters *via* CRISPR/Cas have successfully altered the transcription of mRNAs in several studies (Rodriguez-Leal et al., 2017; Shi et al., 2017; Zeng et al., 2020; Wang et al., 2021; Liu et al., 2021a; Song et al., 2022), providing a useful strategy. Unlike the extensive studies on transcriptional regulations, the mechanism of protein translation has been less studied. Although translational enhancers have been discovered for decades and successfully applied to many transgene studies, the underlying mechanisms are still not well understood that greatly hinders their applications with genome editing. Nevertheless, numerous studies have shown that the inserting of such enhancers into the 5’UTR of transgenic vectors can greatly increase the expression of target proteins (Gallie, 2002; Zou et al., 2003; De Amicis et al., 2007; Sugio et al., 2008; Kanoria and Burma, 2012). And our study here demonstrated that they could also enhance the expression of endogenous genes when precise knock-in to the 5’UTRs, providing a new approach for the up-regulation of target genes.

When we screened the enhancers using rice protoplasts, we found that AMVE has the strongest enhancement effect, and the rice-derived ADHE1 also functions well. However, the ones previously reported to work well in dicots (e.g. Omega) had a much weaker effect, indicating that translational enhancers may have different effects among species. And we may be able to find better enhancers from endogenous UTRs in the future. We also proved that *in-locus* inserting enhancer sequences into 5’UTRs would not affect the transcription of target genes. We think that the enhancement effect is caused by a post-transcriptional mechanism, such as facilitating the initiation of translation or stabilizing post-transcriptional mRNA. And it would be interesting to conduct an in-depth study of this mechanism.

Compared with transcriptional regulations, such translational enhancement has several drawbacks. First, due to the lack of antibodies, it is difficult to quantify the protein level for many genes (e.g. *SKC1*). It would be a big problem if one cannot evaluate the consequence of genome editing. Second, AMVE is the strongest enhancer among the 12 candidates, and up to 10.4-times improvement was found for *SKC1*. However, sometimes we may need a stronger enhancement for breeding, and further screening for better enhancers is recommended in the future. However, we found that the effects of translation enhancers in protoplast-based luciferase reporter were not exactly the same as those of endogenous genes in rice plant. We believe that the flanking sequence of the target gene may also have an impact on protein translation, and that future optimization of reporting systems is needed to accurately evaluate translation regulation. Lastly, it could be found that the translational enhancement differs to some extent among genes. We speculate that this may be affected by the original translation efficiency or expression level of the gene like the cis-elements for transcription regulation. For genes that do not have room for translational optimization, the enhancement may be limited. Despite the success of the two cases on *WRKY71* and *SKC1*, evaluations on other genes would provide more information in the future. Off-target editing is another concern for plant breeding. We tested whether this insertion strategy could induce off-target editing at these predicted off-target sites and no mutation was detected (**Supplementary Table 5**).

In conclusion, we screened a functional enhancer AMVE for translational regulation in rice. Targeted knock-in of AMVE into the 5’UTRs of *WRKY71* and *SKC1* successfully improved the protein abundance, resulted in expected trait improvement. It is the first study that using translational enhancer for such *in-locus* activation of target genes, providing a new strategy to manipulate protein translation. With the development of genome editing technologies, *in-locus* gene activation mediated by editing of regulatory elements will become easier over time, making this strategy a useful method for breeding.

## Data availability statement

The raw data supporting the conclusions of this article will be made available by the authors, without undue reservation.

## Author contributions

YL and RS designed the study. RS, YT, and QY performed the most experiments. QY, DZ, XZ, and XL performed rice transformation. CD and XC genotyped the transgenic lines. RS, YL, YT, and J-KZ wrote and revised the manuscript. All authors contributed to the article and approved the submitted version.
