# Supplementary material for: Targeted insertion of regulatory elements enables translational enhancement in rice
Source: Front Plant Sci. 2023 Mar 31;14:1134209. doi: 10.3389/fpls.2023.1134209 (PMC10102426; doi:10.3389/fpls.2023.1134209)
Supplement: Supplementary file 1 [file DataSheet_1.pdf]

Supplementary Material for

Targeted insertion of regulatory elements enables translational enhancement in rice

Rundong Shen[1,3,5†], Qi Yao[1,2,6†], Dating Zhong[1,2,6†], Xuening Zhang[1,2,6], Xinbo Li[1,3,5], Chao Dong[3,5], Xuesong Cao[1], Yifu Tian[1,3,5*], Jian-Kang Zhu[1,3,4,5*] and Yuming Lu[1,2*]

Supplementary Figures and Tables

Supplementary Figure 1 | Sequence of the dual-luciferase reporter plasmid pDLUC01 **(A)** and the candidate enhancers **(B)**.

Supplementary Figure 2 | Sequence of the dual-luciferase reporter plasmid pDLUC02-WRKY71 **(A)** and pDLUC02-SKC1 **(B)**.

Supplementary Figure 3 | Immunoblot for SKC1 using anti-SKC1 antibodies.

Supplementary Table 1 | Summary of transgenic rice generated in this study.

Supplementary Table 2 | Segregation of AMVE knock-in mutants in the T2 generation.

Supplementary Table 3 | Primers used in this study.

Supplementary Table 4 | Mutants used in this study.

Supplementary Table 5 | Editing frequency at predicated off-target sites.

References

A

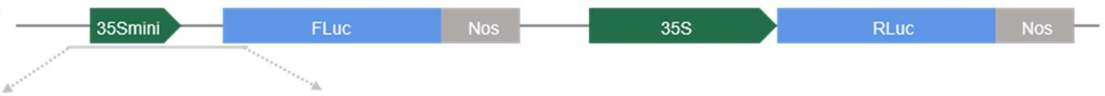

>pDLuc01 (35Smin-Enhancer-FLuc)

TATGACCATGATTACGCCAAGCTTGCATGCCTGCAGGATCTCCACTGACGTAAGGGATGACGCACAATCCCACTATCCT
TCGCAAGACCCTTCCTCTATATAAGGAAGTTCATTTCATTTGGAGAGAACACGGGGGACGGTACCNNNNNNNNNNNNN
NNNNNNNNNNNNATGGAAGACGCCAAAAACATAAAGAAAGGCCCGGCGCCATTCTATCCTCTAGAGGATGGAACCGCT
GGAGAGCAACTGCATAAGGCTATGAAGAGATACGCCCTGGTTCCTGGAACAATTGCTTTTACAGATGCACATATCGA

B

Enhancers	Source	Sequence (5'-3')	Ref
AMVE	AMV	ATAGATACCTCGAGTTTTTATTTTTAATTTTCTTTC AAATACTTCCATCCCTTTTGC	(Jobling and Gehrke, 1987; Kanoria and Burma, 2012)
Omega	TMV	ACCTCGAGTATTTTTACAACAATTACCAACAACAA CAAACAACAAACAACATTACAATTACTATTTACAA TTACA	(Gallie and Walbot, 1992; Gallie, 2002; De Amicis et al., 2007; Kanoria and Burma, 2012)
ADHE	Rice	GTGAATTCCAAGCAACGAACTGCGAGTGATTCAA GAAAAAAGAAAACCTGAGCTTTCGATCTAT	(Sugio et al., 2008)
OspsbA	Rice	AACAAGCCTTCTATTATCTTTCTAGTTAATACGTG TGCTTGGGAGTCCTTGCAATTTGAATAAACCAAG ATCTTA	(Zou et al., 2003)
NtpsbA	Tobacco	AACAAGCCTTCCATTTTCTATTTTGATTTGTAGAAA ACTAGTGTGCTTGGGAGTCCCTGATGATTAAATA AACCAAGATTTTA	(Zou et al., 2003)
NOS	T-DNA	AGAGTCTCATATTCACTCTCAATC	(Kanoria and Burma, 2012)
SynM	Synthetic	ACACGCTGGAATTCTAGTATACTTTT	(Kanoria and Burma, 2012)
SynJ	Synthetic	ACACGCTGGAATTCTAGTATACTAAA	(Kanoria and Burma, 2012)
MsynJ	Synthetic	ACAGGCGCTATCAATCCGAAGCTAAA	(Kanoria and Burma, 2012)
pSTART	Synthetic	GTATTTTTACAACAATACCAACAACAACAACAACA AACAACATTACAATTACGTATTTCTCTCTCTAGAG GATCCCCGGGTGGTCAGTCCCTT	(De Amicis et al., 2007)
GTX	Murine	CCGGCGGGA	(Chappell et al., 2006)
T7g10	Phage	TTAACTTTA	(Inka Borchers et al., 2012)

Supplementary Figure 1 | Sequence of the dual-luciferase reporter plasmid pDLUC01 **(A)** and the candidate enhancers **(B)**.

A

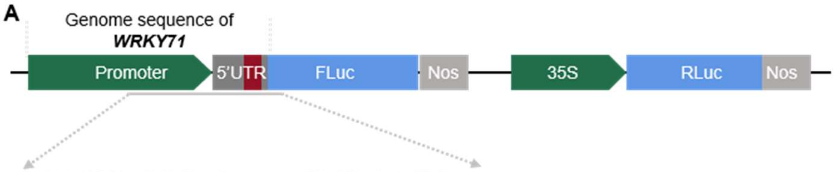

>pDLUC02-WRKY71 (Promoter-5'UTR-AMVE-FLuc)

CTATTTCTATTTTAGGTGGAGCTGAAATTGTTTGGTTGAGCTGGAGCTGTGCCAAACAGGCTCTTTATCTAAAATATAGC
TATTTCTTCTTGTGCTATGTCATATCAAACAATTATAACAATGTTTTATTTAATTTATCTCTATATATTTTATTTCAAATAATC
ATAACCATCTTTTGTTAAATTATAAAACGTCTTATCCACTCAAAAACCCTAGTAATATTTTAAAACGAGTATTATGAAATGA
CGAGATGACAAACACAAGAACTAGAGGGATGTGGTTTTTATTTTTGTTTTAAAAAATAATAATTCAGTTGGCGCCGCTAG
CTGTTCCGGTCACCCTCCAGTCTCCTTCCGATTCTTCCGCCACATCCGCATGCTCGAATTCCCCCTCCCTCCATTTCTTT
ACTCATTTCCCTTCCCCTCCGCTTCCTAATCCTGCGTGACCCCAGACGACGACGACGACGAGTACACATGCGTTTGAGC
GCGACATCCCCGCGCGCGCCTTCTTCTTCCTCTTCCTCCCTCCTCCCATGACTAGACCATCCTCGCCATATTCCCGCCT
CTCTATATATATTAGGGCTCAGCTCCTCCTCTCACCTCGTCTCCTCCTCTCCTCTCTTCTCTCCTTGGCTCAGCTTGACA
TCGTCGGAGCAGCAGAAAAGTTTGCTCGATCGAGCTAGCTAACTAGGTCGTCGGATCAGTGATAAATCGTTCGCATAGA
TACCTCGAGTTTTTATTTTTAATTTTCTTTCAAATACTTCCATCCCTTTTGCCGATGGAAGACGCCAAAAACATAAAGAAA
GGCCCGGCGCCATTCTATCCTCTAGAGGATGGAACCGCTGGAGAGCAACTGCATAAGGCTATGAAGAGATACGCCCTG
GTTCCTGGAACAATTGCTTTTACAGATGCACATATCGA

B

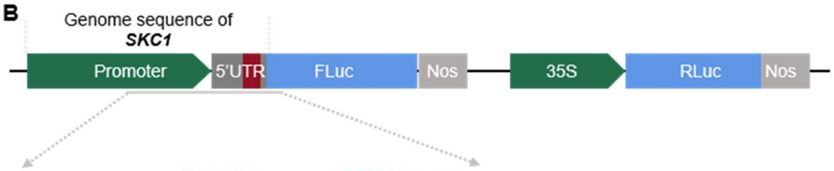

>pDLUC02-SKC1(Promoter-5'UTR-AMVE-FLuc)

CTGCTACAAGAGTATACTAGTTCTCCTCCACTGTGTTCTCTTCAAAAAAAAAAAGAGTATACTAGTTCTAAAATTTTAAAG
TAACAAATTGCAAGTTATAAACATATATGAAGATCTTCGGTAGTTTACATATATGAAGCTTTTAGGATATATATGATATATC
AGTTTAGAAATGAAGGAGGTGACTATATCATCTCAATACATGGAAACTAACTTTGGTGATGATTTACGCACATGAGACAA
AGCATTGCCTGAATCTCGATATATATACAATGTTGGTCTATTTATTAATTGGTGGCTAGTAGTGTTCATCAGATCCTAGAT
GGTACTGTACGTACATGCTAGTTATAAGTTATTGGTGGCTTCAGTTGTTTTCATATATGTGCTACTAGATACTGTTGACGT
CGACGCAGACACTTGTAGCATTAGATTTCATTGTTTTGTCCATAAATATGAAGTCCTATTTGTTGAACTTGATCTCAAATT
TACATCATATAATATATATGTGCCACGAGTGCCCTTGGTGCAATAGCTTTCTGAATGATTGCACTGAGCACAATCCAAAG
ATAAGTATAAAAAAATCGATATACCTCCTGCTGTTGCTCTTGTTCACTTGTGCTCACAGCACCTGGTAACCCCACATACA
GTGATCACACAACAAACAATATCTGTTGTTTGTGTATAAATAGCCCTAACATGGCTTCCTAATTCCTATAAACAACAAGTG
TGAGAAAACCCAAGAACGAAACAATAGATACCTCGAGTTTTTATTTTTAATTTTCTTTCAAATACTTCCATCCCTTTTGCCA
TAGGAGAGAAATGGAAGACGCCAAAAACATAAAGAAAGGCCCGGCGCCATTCTATCCTCTAGAGGATGGAACCGCTGG
AGAGCAACTGCATAAGGCTATGAAGAGATACGCCCTGGTTCCTGGAACAATTGCTTTTACAGATGCACATATCGA

Supplementary Figure 2 | Sequence of the dual-luciferase reporter plasmid pDLUC02-WRKY71 **(A)** and pDLUC02-SKC1 **(B)**.

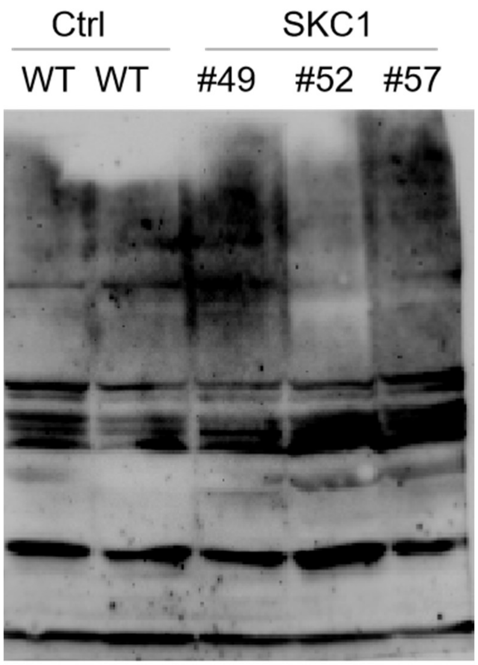

Supplementary Figure 3 | Immunoblot for SKC1 using anti-SKC1 antibodies. Total protein was extracted from leaves of wildtype (WT) and SKC1 mutant plants.

Supplementary Table 1 | Summary of transgenic rice generated in this study

Gene name	Locus	5'UTR (bp)	sgRNA sequence (5'-3')	Number of T0 plants	Number of AMVE knock-in mutants		
					Total (%)	Forward (%)	Vailded* (%)
WRKY71	LOC4328512	120	AGTGATAAATCGTTCGCCGA**TGG**	60	11 (18.3)	5 (8.3)	3 (5.0)
SKC1	LOC4327757	162	AACCCAAGAACGAAACACAT**AGG**	119	32 (26.9)	16 (13.4)	6 (5.0)

* Expected T0 plants with forward and seamless inserted enhancers

Supplementary Table 2 | Segregation of AMVE knock-in mutants in the T2 generation

Mutant lines	Number of T2 plants				Segregation ratio
	Total	Wildtype	Heterozygote	Homozygote	
SKC1#49	86	23	42	21	1:1.8:0.9
SKC1#52	65	15	32	18	1:2.1:1.2
SKC1#57	82	23	40	19	1:1.7:0.8

Supplementary Table 3 | Primers used in this study

Primer name	Primer sequence (5'-3')	Application
AMVE-UP	CATAGATACCTCGAGTTTTTATTTTTAATTTTCTTTCAAATACTTCCATCCCTTTTGCC	Construction of dual-luciferase plasmids
AMVE-LW	CATGGGCAAAAGGGATGGAAGTATTTGAAAGAAAATTAAAAATAAAAACTCGAGGTATCTATGGATC	
ADHE-UP	CGTGAATTCCAAGCAACGAACTGCGAGTGATTCAAGAAAAAAGAAAACCTGAGCTTTCGATCTATC	
ADHE-LW	CATGGATAGATCGAAAGCTCAGGTTTTCTTTTTTCTTGAATCACTCGCAGTTCGTTGCTTGGAATTCACGGATC	
Nos-UP	CAGAGTCTCATATTCACTCTCAATCC	
Nos-LW	CATGGGATTGAGAGTGAATATGAGACTCTGGATC	
SynM-UP	CACACGCTGGAATTCTAGTATACTTTTC	
SynM-LW	CATGGAAAAGTATACTAGAATTCCAGCGTGTGGATC	
SynJ-UP	CACACGCTGGAATTCTAGTATACTAAAC	
SynJ-LW	CATGGTTTAGTATACTAGAATTCCAGCGTGTGGATC	
MsynJ-UP	CACAGGCGCTATCAATCCGAAGCTAAAC	
MsynJ-LW	CATGGTTTAGCTTCGGATTGATAGCGCCTGTGGATC	
OspsbA-UP	CAACAAGCCTTCTATTATCTTTCTAGTTAATACGTGTGCTTGGGAGTCCTTGCAATTTGAATAAACCAAGATCTTAC	
OspsbA-LW	CATGGTAAGATCTTGGTTTATTCAAATTGCAAGGACTCCCAAGCACACGTATTAACTAGAAAGATAATAGAAGGCTTGTTGGATC	
pSTART-UP	CGTATTTTTACAACAATACCAACAACAACAACAACAAACAACATTACAATTACGTATTTCTCTCTCTAGAGGATCCCCGGGTGGTCAGTCCCTTC	
pSTART-LW	CATGGAAGGGACTGACCACCCGGGGATCCTCTAGAGAGAGAAATACGTAATTGTAATGTTGTTTGTTGTTGTTGTTGTTGGTATTGTTGTAAAAATACGGATC	
GTX-UP	CCCGGCGGGAC	
GTX-LW	CATGGTCCCGCCGGGGATC	

NtpsbA-UP	CAACAAGCCTTCCATTTTCTATTTTGATTTGTAGAAAACTAGTGTGCT TGGGAGTCCCTGATGATTAAATAAACCAAGATTTTAC	Construction of dual-luciferase plasmids
NtpsbA-LW	CATGGTAAAATCTTGGTTTATTTAATCATCAGGGACTCCCAAGCACAC TAGTTTTCTACAAATCAAAATAGAAAATGGAAGGCTTGTTGGATC	
T7g10-UP	CTTAACTTTAC	
T7g10-LW	CATGGTAAAGTTAAGGATC	
Omega -UP	CACCTCGAGTATTTTTACAACAATTACCAACAACAACAAACAACAAAC AACATTACAATTACTATTTACAATTACAC	
Omega -LW	CATGGTGTAATTGTAAATAGTAATTGTAATGTTGTTTGTTGTTTGTTGT TGTTGGTAATTGTTGTAAAAATACTCGAGGTGGATC	
sgWRKY-UP	TGTGTGGTGATAAATCGTTCGCCGA	Construction of CRISPR/Cas9 plasmids
sgWRKY-LW	AAACTCGGCGAACGATTTATCACCA	
sgSKC-UP	TGTGTGACCCAAGAACGAAACACAT	
sgSKC-LW	AAACATGTGTTTCGTTCTTGGGTCA	
PWRKY-F1	TTGGCTCAGCTTGACATCGT	Amplification of target sites or insert fragments
PWRKY-R1	AGCACCTTGGGCTTAACCATG	
PSKC-F1	CCCACATACAGTGATCACACA	
PSKC-R1	TACAGCAGTTGGAGCCAGAA	
PWRKY-F2	CCCTCCAGTCTCCTTCCGATT	
PSKC-F2	TGGCTAGTAGTGTTCATCAGAT	
AMVE-MR	GTATTTGAAAGAAAATTAAAA	
AMVE-MF	TTATTTTTAATTTTCTTTCAA	
FLuc-qF	GGATTACAAGATTCAAAGTGCG	qPCR for *FLuc*
FLuc-qR	TGATACCTGGCAGATGGAAC	
RLuc- qF	CATGGGATGAATGGCCTGATATTG	qPCR for *RLuc*
RLuc- qR	GATAATGTTGGACGACGAACTTC	

Actin1-qF	CATTGGTGCTGAGCGTTTC	qPCR for *Actin1*
Actin1-qR	GTACCACCACTGAGAACGATG	
WRKY71-qF	GGCGGCGTCGGAGATGATCAG	qPCR for *WRKY71*
WRKY71-qR	CCGCCTTGAAGCTTGGGTCAC	
SKC1-qF	TGGCTCCAACTGCTGTACTTCC	qPCR for *SKC1*
SKC1-qR	ACCATGCTCGACACCGT	
OFF-F1	CCACGGAACGAGATCCCAGC	Amplification of off-target sites
OFF-R1	CCCTCGCCACCGAGTTGATT	
OFF-F2	AGGCGAATGCCCGATCCAAGA	
OFF-R2	TGGCGGAGATTTGGGGGAGA	
OFF-F3	ACCGCTCGTGCACTTCACTC	
OFF-R3	GCGTGTGCAAACAGTGCCAT	
OFF-F4	AGCATCTAGACGACGCAGCC	
OFF-R4	GGTTACATAACAATGGGAGGA	
OFF-F5	GCGGTGGGAAAAGGAGGAGG	
OFF-R5	GACGAGACGATGACTCGGCG	
OFF-F6	TGCATGAGTTCATCTGGGCAA	
OFF-R6	TGTGTTTGGTTCCTGAGATC	
OFF-F7	GGTTGGTTCTGGTCCTCGCC	
OFF-R7	CGCCATGTCAGCCAAAACCG	
OFF-F8	TAGTGTGCCCCTCCCTGCTT	
OFF-R8	CGAGCTTCCCTTAGGGTGCG	
OFF-F9	TGGAACTCAGGAAGTGAATTTCGG	
OFF-R9	ATGGTCAAACGTTATACGAAAAGTC	
OFF-F10	TAAGCGCCACGTCAGCCAAA	
OFF-R10	CGAGAATCAGGAGCAATTGATGACA	

Supplementary Table 4 | Mutants used in this study

<table>
<tr><th>Mutants name</th><th>Mutation type</th><th>Genotype (5'-3')</th></tr>
<tr><td>WRKY71#KI05</td><td>WT</td><td>agctaactaggtcgtcggatcagtgataaatcgttcgc | cgATGGATCCGT</td></tr>
<tr><td>WRKY71#KI04</td><td>Forward AMVE insertion</td><td>agctaactaggtcgtcggatcagtgataaatcgttcgcATAGATACCTCGAGTTTTTATTTTTAAT
TTTCTTTCAAATACTTCCATCCCTTTTGCcgATGGATCCGT</td></tr>
<tr><td>WRKY71#KI07</td><td>Forward AMVE insertion</td><td>agctaactaggtcgtcggatcagtgataaatcgttcgcATAGATACCTCGAGTTTTTATTTTTAAT
TTTCTTTCAAATACTTCCATCCCTTTTGCcgATGGATCCGT</td></tr>
<tr><td>WRKY71#KI14</td><td>Forward AMVE insertion</td><td>agctaactaggtcgtcggatcagtgataaatcgttcgcATAGATACCTCGAGTTTTTATTTTTAAT
TTTCTTTCAAATACTTCCATCCCTTTTGCcgATGGATCCGT</td></tr>
<tr><td>SKC#KI51</td><td>WT</td><td>tataaacaacaagtgtgagaaaacccaagaacgaaaca | cataggagagaaATGAGTTCTC</td></tr>
<tr><td>SKC#KI49</td><td>Forward AMVE insertion</td><td>tataaacaacaagtgtgagaaaacccaagaacgaaacaATAGATACCTCGAGTTTTTATTTTT
AATTTTCTTTCAAATACTTCCATCCCTTTTGCcataggagagaaATGAGTTCTC</td></tr>
<tr><td>SKC#KI52</td><td>Forward AMVE insertion</td><td>tataaacaacaagtgtgagaaaacccaagaacgaaacaATAGATACCTCGAGTTTTTATTTTT
AATTTTCTTTCAAATACTTCCATCCCTTTTGCcataggagagaaATGAGTTCTC</td></tr>
<tr><td>SKC#KI57</td><td>Forward AMVE insertion</td><td>tataaacaacaagtgtgagaaaacccaagaacgaaacaATAGATACCTCGAGTTTTTATTTTT
AATTTTCTTTCAAATACTTCCATCCCTTTTGCcataggagagaaATGAGTTCTC</td></tr>
</table>

Supplementary Table 5 | Editing frequency at predicated off-target sites

Site	Chromosome	Position	Guide-PAM sequence	Mismatch numbers	Editing efficiency
Off-target 1	7	-2936072	GGTGATTAATCGGTCGCCGA**TGG**	3	0
Off-target 2	9	-7225044	AGTGGTGAATGGTTCGCTGA**TGG**	4	0
Off-target 3	3	+34550431	AGTGATATATCGATCGTCCA**TGG**	4	0
Off-target 4	1	+42324424	AGTGATCGATCGATCGCGGA**AGG**	4	0
Off-target 5	2	+26412400	AGTTATAAATCATTCGACCA**TAG**	4	0
Off-target 6	6	+27784911	ATCCCAAGAACAGAACACAT**GGG**	3	0
Off-target 7	3	-25970626	AACCGAAAAACCAAACACAA**CGG**	4	0
Off-target 8	7	-24917595	AACCCAAGGAAGAAACAAAA**AGG**	4	0
Off-target 9	6	-27819350	CACACAAGAAAGAAAAACAT**AAG**	4	0
Off-target 10	10	-22599673	AACCCAAAAACCAGACACAA**CGG**	4	0

References

Chappell, S.A., Dresios, J., Edelman, G.M., and Mauro, V.P. (2006). Ribosomal shunting mediated by a translational enhancer element that base pairs to 18S rRNA. *Proceedings of the National Academy of Sciences of the United States of America* 103, 9488-9493.

De Amicis, F., Patti, T., and Marchetti, S. (2007). Improvement of the pBI121 plant expression vector by leader replacement with a sequence combining a poly(CAA) and a CT motif. *Transgenic Res* 16, 731-738.

Gallie, D.R. (2002). The 5'-leader of tobacco mosaic virus promotes translation through enhanced recruitment of eIF4F. *Nucleic acids research* 30, 3401-3411.

Gallie, D.R., and Walbot, V. (1992). Identification of the motifs within the tobacco mosaic virus 5'-leader responsible for enhancing translation. *Nucleic acids research* 20, 4631-4638.

Inka Borchers, A.M., Gonzalez-Rabade, N., and Gray, J.C. (2012). Increased accumulation and stability of rotavirus VP6 protein in tobacco chloroplasts following changes to the 5' untranslated region and the 5' end of the coding region. *Plant biotechnology journal* 10, 422-434.

Jobling, S.A., and Gehrke, L. (1987). Enhanced translation of chimaeric messenger RNAs containing a plant viral untranslated leader sequence. *Nature* 325, 622-625.

Kanoria, S., and Burma, P.K. (2012). A 28 nt long synthetic 5'UTR (synJ) as an enhancer of transgene expression in dicotyledonous plants. *BMC biotechnology* 12, 85.

Sugio, T., Satoh, J., Matsuura, H., Shinmyo, A., and Kato, K. (2008). The 5'-untranslated region of the Oryza sativa alcohol dehydrogenase gene functions as a translational enhancer in monocotyledonous plant cells. *Journal of bioscience and bioengineering* 105, 300-302.

Zou, Z., Eibl, C., and Koop, H.U. (2003). The stem-loop region of the tobacco psbA 5'UTR is an important determinant of mRNA stability and translation efficiency. *Molecular genetics and genomics : MGG* 269, 340-349.
